# A Comparative Study of the Influence of Nitrogen Content and Structural Characteristics of NiS/Nitrogen-Doped Carbon Nanocomposites on Capacitive Performances in Alkaline Medium

**DOI:** 10.3390/nano11071867

**Published:** 2021-07-20

**Authors:** Mohamed M. Abdelaal, Tzu-Cheng Hung, Saad Gomaa Mohamed, Chun-Chen Yang, Huei-Ping Huang, Tai-Feng Hung

**Affiliations:** 1 Battery Research Center of Green Energy, Ming Chi University of Technology, 84 Gungjuan Rd., Taishan District, New Taipei City 24301, Taiwan; mohamedbec@yahoo.com (M.M.A.); u07137023@mail2.mcut.edu.tw (T.-C.H.); ccyang@mail.mcut.edu.tw (C.-C.Y.); cindy118036@mail.mcut.edu.tw (H.-P.H.); 2 Tabbin Institute for Metallurgical Studies (TIMS), Tabbin, Helwan 109, Cairo 11421, Egypt; sgmmohamed@gmail.com; 3 Department of Chemical Engineering, Ming Chi University of Technology, 84 Gungjuan Rd., Taishan District, New Taipei City 24301, Taiwan; 4 Department of Chemical and Materials Engineering, Chang Gung University, 259 Wenhua 1st Rd., Guishan District, Taoyuan 33302, Taiwan

**Keywords:** transition metal sulfides, polymer-derived nitrogen-doped carbon, microwave-assisted synthesis, supercapacitors, alkaline electrolyte

## Abstract

Supercapacitors (SCs) have been regarded as alternative electrochemical energy storage devices; however, optimizing the electrode materials to further enhance their specific energy and retain their rate capability is highly essential. Herein, the influence of nitrogen content and structural characteristics (i.e., porous and non-porous) of the NiS/nitrogen-doped carbon nanocomposites on their electrochemical performances in an alkaline electrolyte is explored. Due to their distinctive surface and the structural features of the porous carbon (A-PVP-NC), the as-synthesized NiS/A-PVP-NC nanocomposites not only reveal a high wettability with 6 M KOH electrolyte and less polarization but also exhibit remarkable rate capability (101 C/g at 1 A/g and 74 C/g at 10 A/g). Although non-porous carbon (PI-NC) possesses more nitrogen content than the A-PVP-NC, the specific capacity output from the latter at 10 A/g is 3.7 times higher than that of the NiS/PI-NC. Consequently, our findings suggest that the surface nature and porous architectures that exist in carbon materials would be significant factors affecting the electrochemical behavior of electrode materials compared to nitrogen content.

## 1. Introduction

Recent technological innovations have increased the widespread application of various electrochemical energy storage (EES) fields. Furthermore, scientists have developed numerous EES devices to store more energy with a long-life cycle. Among the existing EES devices, there is a complementary relationship between metal-ion batteries (MIBs) and supercapacitors (SCs) since the former has a high energy density, but the latter can deliver extreme power density [1,2,3,4,5]. It is recognized that increasing the power energy of the MIBs to as high as that of the SCs is intrinsically challenging, owing to the insertion/extraction of the metal ions from the corresponding electrode materials being principally essential, that is, the energy storage mechanism [6,7,8,9,10]. On the contrary, it has been demonstrated that several electrode materials that followed a redox reaction can provide more capacitances as compared with the traditionally used carbonaceous materials [11,12,13].

So far, the metal sulfides (MSs)-based nanostructures have attracted much attention as one of the promising battery-type electrodes because of their remarkably specific capacitance [14,15]. To ensure their outstanding rate capability, the rational design and synthesis for formulating a series of MSs/carbon nanocomposites by combining various carbonaceous materials have also been reported recently [16,17,18,19]. The advantages of carbonaceous materials include not only good electronic conductivity but also their availability, light weight, thermal properties, chemical inertness, and so forth [20,21,22]. In addition to the pristine, it is noticed that employing nitrogen-doped carbon as a conductive species can further enhance the rate performance as well, which is attributed to the heteroatoms that exist within the carbonaceous materials, which enable high electronic conductivity [23,24,25].

For synthesizing the MSs/carbon nanocomposites, it is suggested that bottom-up procedures, such as hydro/solvothermal reactions [26,27] and aerosol-assisted spray pyrolysis [28], be preferred since the MSs nanocrystals could be uniformly distributed onto the carbonaceous materials. However, the methods described earlier are usually time-consuming and energy-wasting processes as compared with a microwave reactor. Unlike the conventional heat-transportation mode, the energy required for growing the nanocrystals can be efficiently and homogeneously diffused to the precursors through microwave irradiation [29,30,31]. Therefore, the samples thus obtained are similar to those synthesized by previous methods and can even be completed in a shorter time or at a lower temperature [32,33].

In this study, two polymer-derived nitrogen-doped carbon materials with different nitrogen contents and textural properties (i.e., porous and non-porous) were first prepared using a pyrolysis procedure under an Ar atmosphere. The nitrogen atoms presented in the doped carbon originated from their parent polymers. The as-prepared carbon materials were identified utilizing powder X-ray diffractometry (PXRD), Raman spectroscopy, transmission electron microscopy (TEM), a nitrogen adsorption–desorption analyzer, and elemental analysis measurement. Subsequently, a microwave-assisted approach was adopted to synthesize the NiS/nitrogen-doped carbon nanocomposites for exploring the influence of nitrogen content and structural characteristics on the capacitive performance of alkaline supercapacitors. The crystalline structure and morphology of each NiS/nitrogen-doped carbon nanocomposite were confirmed by refining the corresponding PXRD pattern and TEM observation. The wettability between each NiS/nitrogen-doped carbon nanocomposite and electrolyte was statically checked in ambient conditions. The electrochemical performances were evaluated in an N_2_-saturated alkaline electrolyte using cyclic voltammetry (CV), the galvanostatic charge-discharge (GCD) procedures and electrochemical impedance spectroscopy (EIS). To the best of our knowledge, the comparison between the impact of both nitrogen content and the structural characteristics of the MSs/nitrogen-doped carbon nanocomposites on the electrochemical performances of SCs has not been discussed at the same time. The findings in this study would offer an alternative point of view for designing MSs/nitrogen-doped carbon nanocomposites with extensive applications.

## 2. Materials and Methods

### 2.1. Chemicals

All reagents, including polyvinylpyrrolidone (PVP, (C_6_H_9_NO)n, average MW 1,300,000, Sigma-Aldrich, St. Louis, MO, USA), polyimide (PI-2, SHIFENG TECHNOLOGY CO., LTD., Tainan, Taiwan), nickel (II) nitrate hexahydrate (Ni(NO_3_)_2_·6H_2_O, 98%, Alfa Aesar, Heysham, England), thioacetamide (CH_3_CSNH_2_, 98%, Alfa Aesar, Heysham, England), ascorbic acid (C_6_H_8_O_6_, ACS reagent, Honeywell Fluka™, Charlotte, NC, USA), ethylene glycol (HOC_2_H_4_OH, Baker Analyzed™ reagent, J.T. Baker, Center Valley, PA, USA), ethyl alcohol (C_2_H_5_OH, ≥99.5%, Sigma-Aldrich, St. Louis, MO, USA), potassium carbonate (anhydrous, K_2_CO_3_, 99%, Alfa Aesar, Heysham, England), potassium chloride (KCl, ACS reagent, Sigma-Aldrich, St. Louis, MO, USA), potassium hydroxide (KOH, 99.99%, Sigma-Aldrich, St. Louis, MO, USA), carbon black (Super P^®^, Timcal Ltd., Bodio, Switzerland) and Nafion^®^ perfluorinated resin solution (5 wt. %, Sigma-Aldrich, St. Louis, MO, USA) were employed without further purification. Deionized (DI) water produced from a Milli-Q^®^ Integral water purification system (Millipore Ltd., Burlington, MA, USA) was used throughout the experiments.

### 2.2. Preparation of Polymer-Derived Nitrogen-Doped Carbon Materials

To prepare the polyimide-derived carbon (PI-NC) sample, 5 g of PI powders were thermally decomposed in the tube furnace at 900 °C for 8 h under an argon atmosphere with a flow rate of 200 mL/min. The as-prepared PI-NC powders were collected when they were naturally cooled down to room temperature and thoroughly ground using pestle and agate mortar. As for the porous carbon, 4 g of polyvinylpyrrolidone powders were well-dissolved in the DI water (solution 1). Afterwards, the same mass ratio of the KCl and the K_2_CO_3_ aqueous solutions were sequentially added to solution 1. Here, the former was adopted as the template, while the latter served as the activator [34]. The viscous mixture had been continuously stirred for 3 h before it was completely dried in the oven at 120 °C for 12 h. The resultant residues were then pyrolyzed in the tube furnace using the same temperature, atmosphere and flow rate as the PI-NC but only for 2 h. Following this, repeatedly rinsing with DI water to remove the KCl, drying and grinding procedures were carried out in order. At this point, the loose powders—the so-called activated polyvinylpyrrolidone-derived carbon (A-PVP-NC)—can be obtained eventually.

### 2.3. Microwave-Assisted Synthesis of NiS/Nitrogen-Doped Carbon Nanocomposites

For synthesizing the NiS/nitrogen-doped carbon nanocomposites, the typical processes were described as follows. Firstly, the nitrogen-doped carbon powders (0.05 g A-PVP-NC or 0.5 g of PI-NC) were homogenously dispersed within the 20 mL of HOC_2_H_4_OH by ultrasonication for 1 h (solution 1). Another 20 mL of the emerald green aqueous solution composed of the ingredients (equal molar of Ni(NO_3_)_2_·6H_2_O, CH_3_CSNH_2_, and C_6_H_8_O_6_) were then mixed with solution 1 under magnetically stirring for 1 h. Subsequently, the precursor solution was carefully transferred to a microwave reactor (Discover^®^ System, CEM Corporation, Matthews, NC, USA). The output power, reaction temperature and period were set as 100 W, 150 °C and 1 h, respectively. During the microwave heating procedure, the precursor solution was continuously stirred to ensure a homogeneous reaction. The product was collected by filtration and repeated rinsing with DI water, C_2_H_5_OH and drying in the air (60 °C for 12 h). The as-synthesized NiS/A-PVP-NC or NiS/PI-NC were acquired after being thoroughly ground using pestle and agate mortar.

### 2.4. Characterizations

The crystalline structures of A-PVP-NC, PI-NC, NiS/A-PVP-NC and NiS/PI-NC were identified using a powder X-ray diffractometer (PXRD, Bruker D2 PHASER) with a Cu target (λ = 1.541 Å) that was excited at 30 kV and 10 mA. In addition, the corresponding PXRD patterns of NiS/A-PVP-NC and NiS/PI-NC recorded in the range of 2θ from 10° to 100° at a scanning rate of 2.5 sec/step were further refined via Rietveld analysis using TOPAS 4.2 software (Bruker AXS Inc., Karlsruhe, Germany). The Raman spectrum was collected by a Raman microscope (inVia, Renishaw, UK) equipped with a 633 nm laser source. The elemental mappings were examined by a scanning electron microscope (SEM, S-2600H, Hitachi, Ltd., Tokyo, Japan) connected with energy-dispersive X-ray spectroscopy (XFlash^®^ EDX Detector 3001, Bruker AXS Mikronalysis GmbH, Karlsruhe, Germany). For morphological observations, a transmission electron microscope (TEM, JEM-2100, JEOL Ltd., Tokyo, Japan), operating at an accelerating voltage of 200 kV, was utilized. N_2_ adsorption–desorption isotherm was recorded at 77 K on a surface area and porosity analyzer (ASAP 2020 V3.00, Micromeritics Instrument Corporation, Norcross, GA, USA) after the A-PVP-NC had been degassed in a vacuum at 160 °C for 8 h. An elemental analyzer (FLASH 2000, Thermo Fisher Scientific Inc., Waltham, MA, USA) was applied for determining the percentage of carbon, nitrogen and oxygen within the polymers, nitrogen-doped carbon and NiS/nitrogen-doped carbon nanocomposites. The chemical environments were analyzed with X-ray photoelectron spectroscopy (XPS, PHI 5000 VersaProbe III, ULVAC-PHI, Inc., Kanagawa, Japan) with a beam size of 100 um under Al Kα radiation (λ = 8.3406 Å). Their corresponding spectra were analyzed by the Gaussian–Lorentzian fitting method using an XPSPEAK 4.1 software. The compatibility between each NiS/nitrogen-doped carbon nanocomposite and electrolyte was carefully added to 0.5 mL of 6 M KOH solution in a glass vial that contained 0.1 g of NiS/nitrogen-doped carbon powder, in order to statically check their wettability in ambient conditions.

### 2.5. Electrochemical Measurements

The electrochemical tests throughout this study were conducted in a standard three-electrode system, which was controlled by a multichannel electrochemical workstation (VMP3, Bio-Logic, Seyssinet-Pariset, France) in ambient conditions. To prepare the working electrode, 8 mg of active material (NiS/A-PVP-NC or NiS/PI-NC) and 2 mg of carbon black were well-dispersed within the solution (0.5 mL of C_2_H_5_OH, 0.25 mL of DI water, and 0.25 mL of 5 wt. % Nafion^®^ solution) by ultrasonication for 30 min. Then, 5 µL of the homogenous suspension was added dropwise onto the surface of a glassy carbon electrode (d = 3 mm, #CHI104, CH Instruments, Inc., Austin, TX, USA), resulting in the mass loading of about 0.566 mg/cm^2^ of the active material. A graphite rod and a saturated calomel electrode (SCE, Hg/Hg_2_Cl_2_ (sat. KCl), #CHI150, CH Instruments, Inc.) served as the counter and reference electrodes, respectively. The cyclic voltammograms (CVs), galvanostatic charge-discharge (GCD) profiles, and electrochemical impedance spectroscopy (EIS) spectra were accomplished under an N_2_-saturated 6 M KOH electrolyte. The potential and scanning rates used for CVs were in the range of 0 V and 0.5 V (vs. SCE) and from 10 mV/sec to 100 mV/sec, respectively. Regarding the conditions applied for GCD results, the electrodes were evaluated from the potential of 0 V to 0.4 V (vs. SCE), adopting the current densities of 1 A/g to 10 A/g. The corresponding specific capacity (*C_s_*) can be calculated through the GCD curve according to the equation of *C_s_* (C/g) = *I*Δ*t*/*m*, where *I*, Δ*t*, and *m* represent the current (A), discharge time (sec.) and the mass of the active material (g) [35,36]. The EIS spectra were recorded at open circuit potential (OCP) from 100 kHz to 0.01 Hz with an AC potential amplitude of 5 mV.

## 3. Results

### 3.1. Characterizations of A-PVP-NC and PI-NC

**Figure 1:**
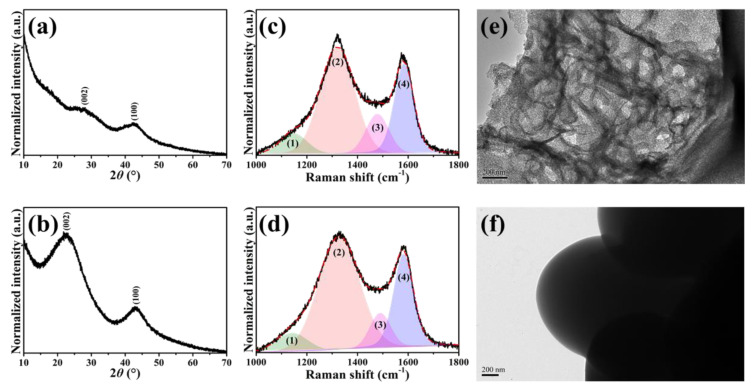
XRD patterns, fitted Raman spectra and low-magnification TEM micrographs of A-PVP-NC (**a**,**c**,**e**) and PI-NC (**b**,**d**,**f**). Scale bars of (**e**,**f**) are 200 nm.

Figure 1 compares the normalized PXRD patterns, fitted Raman spectra and low-magnification TEM micrographs of A-PVP-NC and PI-NC. First of all, two predominant peaks representing the (002) and (100) crystallographic planes of carbon were reflected in Figure 1a,b, confirming the successful preparation of polymer-derived carbon materials with high purity. However, it is obvious that the characteristic peaks of PI-NC were more intense than those of the A-PVP-NC, indicating less crystallinity for the A-PVP-NC. This result might be correlated with the presence of KCl (template) and K_2_CO_3_ (activator), leading to the porous and loosely structural features after high-temperature pyrolysis and rinsing procedures. This phenomenon is typically observed from the activated carbon materials [34].

As for the Raman characterization, two distinct peaks assigned to the D (~1327 cm^−1^) and G (~1583 cm^−1^) bands were undoubtedly displayed in each spectrum (Figure 1c,d). On the other hand, it is known that the intensity ratio of the D and G bands (*I*_D_/*I*_G_) is generally adapted to evaluate the degree of defects in the graphite material. Thus, an increase in the *I*_D_/*I*_G_ ratio suggests decreased graphite integrity, signifying that many defects and/or highly disordered degrees have existed. This is also normally observed in carbon materials containing many functional groups [37,38]. As revealed, the high *I*_D_/*I*_G_ ratio for the A-PVP-NC (1.18) and PI-NC (1.13) could be attributed to the presence of heteroatoms (i.e., N and O) and less crystallinity as evidenced by PXRD patterns. Furthermore, the corresponding Raman spectra were sequentially deconvoluted into four peaks (labeled peaks (1)–(4)) since the integrated area ratio of sp^3^ to sp^2^ (*A_sp_*^3^/*A_sp_*^2^) has been demonstrated to deliver helpful information about the nature of carbon, for example, a low *A_sp_*^3^/*A_sp_*^2^ ratio indicates that a large amount of carbon exists as the sp^2^ type [39,40]. As depicted, the peaks of (2) and (4) are related to sp^2^-type carbon, while the others are associated with sp^3^-type carbon. The calculated values of the *A_sp_*^3^/*A_sp_*^2^ ratio were 0.26 for A-PVP-NC and 0.19 for PI-NC, inferring that the PI-NC exhibited high proportions of sp^2^-type carbons.

Owing to the A-PVP-NC being achieved by incorporating the KCl and K_2_CO_3_ then following through a high-temperature pyrolysis and rinsing procedures, it is reasonably believed that highly porous architectures could be revealed. As expected, numerous hierarchical pores were shown, as seen in Figure 1e. In contrast, a non-porous structure was revealed from the PI-NC (Figure 1f). To accurately determine the classification of pores and specific surface area (SSA) of the A-PVP-NC, the nitrogen adsorption–desorption isotherm measurement was conducted. Figure S1 illustrates a typical isotherm curve, referred to as type I, and a predominant pore diameter less than 2 nm was detected, indicating a microporous characteristic of the A-PVP-NC [34]. Calculated by the Brunauer–Emmett-Teller (BET) method, the SSA value was as high as 1628 m^2^/g. Moreover, it is noticed that the SSA value of micropores was 1483 m^2^/g, whereas that for mesopores was just 52 m^2^/g. Accordingly, the total pore volume collected at P/P_0_ = 0.995 was 0.79 cm^3^/g, while the pore size distributions of ultramicropores, micropores and mesopores were also measured to be 0.25, 0.62, and 0.17 cm^3^/g, respectively (Table S1). The fascinating properties described above would be beneficial for absorbing more electrolytes, thus facilitating ionic transportation and energy storage [41].

To further understand the composition, elemental analyses of A-PVP-NC and PI-NC were carried out to determine the percentage of C, O and N. Prior to the pyrolysis, the nitrogen (oxygen) content was determined to be 11.3% (32.6%) and 6.0% (24.4%) for PVP and PI, respectively. This difference is reasonably ascribed to the molecular weight of the former (1,300,000) being much higher than that of the latter. It is found that the proportion of carbon in both materials was the same, at 86%, whereas that of oxygen is 8.0% and 5.7% in A-PVP-NC and PI-NC in turn. The primary influential factor is the percentage of nitrogen; the value of PI-NC was 3.4%, six times as much as its presence in A-PVP-NC. Kim et al. monitored the pyrolysis reaction of the two employed polymers and discovered that PVP completely decomposes between 350–450 °C, whereas PI pyrolyzes at 550–650 °C [42]. The main reason for the high stability of the PI structure is the presence of resonance between the nitrogen atom and two carbonyl groups in ortho positions in the same ring, while the resonance in PVP is between the nitrogen atom and one carbonyl group [43]. Notably, the pores of PI-NC are sealed by deposited carbon, resulting from the pyrolysis process [44]. Consequently, the surface of PI-NC is non-porous, and it impedes the removal of nitrogen from the structure. On the contrary, the PVP is utilized as a pore-forming agent due to gas emissions, resulting in the elimination of most of the nitrogen products as gases during the pyrolysis process [45]. Moreover, it is also reported that the nitrogen species contained in the carbon precursor/char were preferentially removed during chemical activation with K-based salts [34]. These would support the result of the higher nitrogen percentage for PI-NC after pyrolysis, as opposed to A-PVP-NC. According to the results discussed above, it is rationally anticipated that the A-PVP-NC, thus prepared, could contribute to more homogeneous growth of the NiS and provide sufficient pores as the electrolyte reservoir for enhancing the electrochemical performances more than those of the PI-NC.

### 3.2. Crystalline, Chemical Environmental, Morphological, and Wettability Observations of NiS/A-PVP-NC and NiS/PI-NC

**Figure 2:**
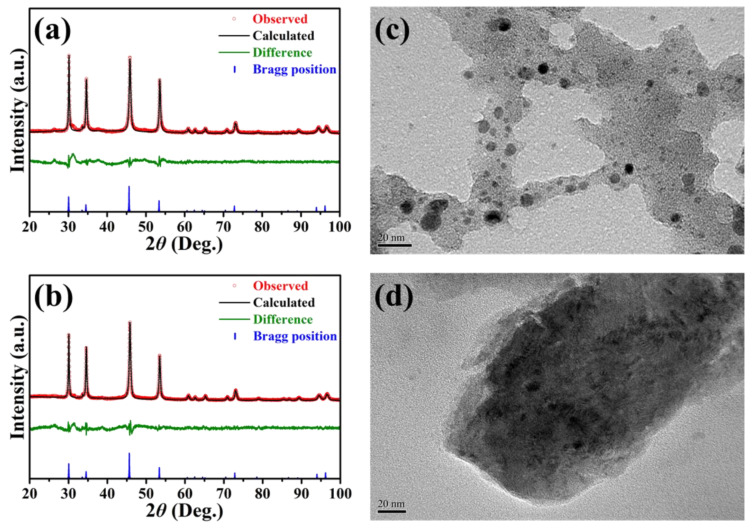
Rietveld refined PXRD patterns and high-magnification TEM micrographs of NiS/A-PVP-NC (**a**,**c**) and NiS/PI-NC (**b**,**d**). Scale bars of (**c**,**d**) are 20 nm.

Figure 2 shows the Rietveld refined PXRD patterns and high-magnification TEM micrographs of NiS/A-PVP-NC and NiS/PI-NC. Given the highly efficient and uniform microwave irritation, both resultant NiS/carbon nanocomposites has good crystallinity and purity (Figure 2a,b), even only with a short reaction period (i.e., 1 h). The predominant peaks located around the 2θ of 30°, 34°, 46°, and 54° were well-matched with the (100), (101), (102) and (110) planes of hexagonal α-NiS (JCPDS No.: 75–0613, space group: P63/mmc, a = b = 3.42 Å, c = 5.30 Å) [46]. In addition, a broad peak at 2θ = 26.3° with less intensity was detected, mainly referring to the (002) plane of carbon materials. Interestingly, another peak corresponding to the (300) plane of NiS@carbon materials appeared at 2θ = 31.4° [47]. These results suggest that the as-prepared carbon materials from two different sources did not contribute to the crystalline phase of NiS.

Because of the high SSA of, and more oxygen in, the A-PVP-NC, the NiS particles with a size of less than 10 nm were deposited onto the A-PVP-NC (Figure 2c). They were even better dispersed in comparison with the NiS/PI-NC, which is revealed in Figure 2d. This distribution would be beneficial for increasing the electrochemically active surface area of NiS for the redox reactions [47]. On the other hand, it is shown that fewer carbon signs were detected by the energy-dispersive X-ray spectroscopy (EDX) mapping in the NiS/PI-NC (Figure S2b2) as compared with the NiS/A-PVP-C (Figure S2a2). This observation would be attributed to the severe agglomeration of the NiS grown onto the PI-NC’s surface, which was consistent with the micrograph shown in Figure 2d. As for the other elements (i.e., N, O, Ni, S), they were distributed throughout the resultant NiS/nitrogen-doped carbon nanocomposites. To clarify the effect of the microwave irritation on the percentage of N and O that existed in the NiS/nitrogen-doped carbon nanocomposites, the EA results were examined. It is observed that the nitrogen content for the NiS/A-PVP-NC and NiS/PI-NC was 0.33% and 3.1%, respectively, showing no significant change as compared to the pristine nitrogen-doped carbon samples (A-PVP-NC: 0.47% and PI-NC: 3.4%). These results reasonably suggest that the nitrogen contents detected in the resultant nanocomposites originated from the A-PVP-NC and PI-NC, implying that no extra nitriding occurred under 150 °C of the microwave irradiations.

**Figure 3:**
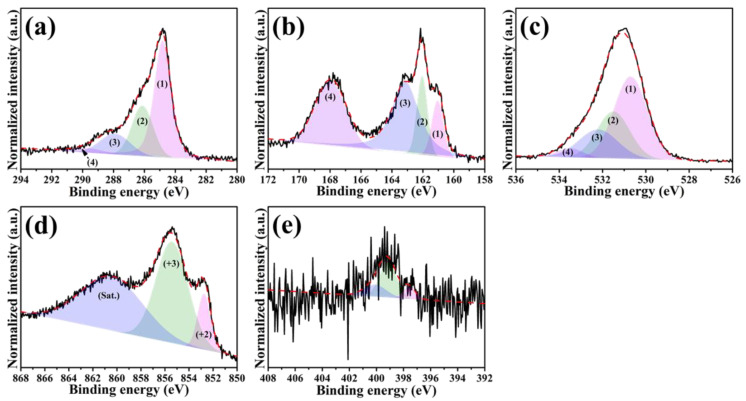
High-resolution XPS spectra of the NiS/A-PVP-NC: (**a**) C 1s ((1) for C=C bond, (2) for C−O bond, (3) for C=O bond, and (4) for O=C−O bond), (**b**) S 2p ((1)–(3) assigned to S^2−^ within the NiS, and (4) obtained from sulfate ions), (**c**) O 1s ((1) and (3) associated with the Ni^3+^OOH and Ni^2+^SO_4_, (2) for O=C−O bond, and (4) for C=O bond), (**d**) Ni 2p and (**e**) N 1s.

To identify detailed information about the chemical environments of our NiS/nitrogen-doped carbon nanocomposites, XPS analysis was conducted, while the corresponding spectra analyzed using the Gaussian–Lorentzian fitting method are illustrated in Figure 3 and Figure S3. It has previously been confirmed that the nitrogen atoms were incorporated into the NiS-based nanocomposites by an elemental analyzer. Here, we did not especially highlight the C-N bonding in C 1s spectra for better reading. From the C 1s spectra plotted in Figure 3a and Figure S3a, a predominant peak assigned for the sp^2^-type carbon with C=C bond (1) appeared at 284.8 eV. Moreover, three peaks (C−O bond (2) at 286.3 eV, C=O bond (3) at 287.9 eV and O=C−O bond (4) at 289.7 eV) deconvoluted from a higher energy level were also observed, which was attributed to the contribution of sp^3^-type carbon [39]. For the S 2p spectrum (Figure 3b), the constitute peaks ((1)~(3)) between the binding energy of 161 eV and 163 eV are assigned to S^2−^ within the NiS. However, more peak intensity and area (around 167.8 eV, peak 4) generated from the NiS/A-PVP-NC were noticed, which is likely due to more sulfate ions resulting from surface oxidation [46]. For the O 1s spectrum (Figure 3c), the peaks at the binding energies of 530.7 eV (1) and 532.2 eV (3) are associated with the Ni^3+^OOH and Ni^2+^SO_4_ [48], whereas the peaks at the binding energies of 531.5 eV (2) and 533.5 eV (4) could be owed to the O=C−O bond and C=O bond, respectively [49]. Due to the surface oxidation evidenced in Figure 3b,c, the mixing valence states (Ni^2+^: 852.7 eV and Ni^3+^: 855.4 eV) were observed from Figure 3d and Figure S3d. Worth mentioning is that the proportion of Ni^3+^ in the NiS/A-PVP-NC is higher than that of the NiS/PI-NC, that is, 82% vs. 53%, which could provide rich redox reactions of NiS [50]. For the N 1s spectrum, the typical fingerprints corresponding to pyridinic-N, pyrrolic-N and graphitic-N were revealed in the 397.6 eV, 399.2 eV and 400.1 eV [51]. The above results not only clearly identify the binding configurations within the NiS/nitrogen-doped carbon nanocomposites, but also help us to better understand the functions of nitrogen and oxygen.

**Figure 4:**
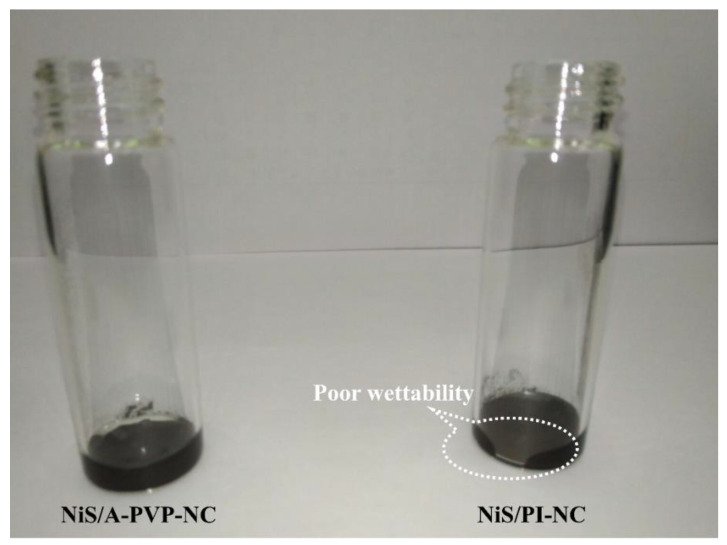
Wettability test of NiS/A-PVP-NC (**left side**) and NiS/PI-NC (**right side**).

As discussed earlier, the hierarchically porous architectures could provide a variety of properties, enlarging the electrochemical performances of the EES devices [52]. Hence, the compatibility between the NiS/nitrogen-doped carbon nanocomposites and alkaline electrolytes is worth confirming. As presented in Figure 4, it is shown that the NiS/A-PVP-NC was wetted immediately, while the NiS/PI-NC resisted the electrolyte, as highlighted by the white circle. Although the high nitrogen content increases wettability [53], the oxygen-containing functional groups and porous structures deliver more significant contributions [41,54,55]. As a result, the NiS-A-PVP-NC possessed superior wettability, with a 6 M KOH electrolyte, than the NiS/PI-NC. This can also be supported by elemental analysis, since the NiS/A-PVP-NC possessed a much higher oxygen content than the NiS/PI-NC, that is, 7.5% vs. 5.1%.

### 3.3. Electrochemical Studies

**Figure 5:**
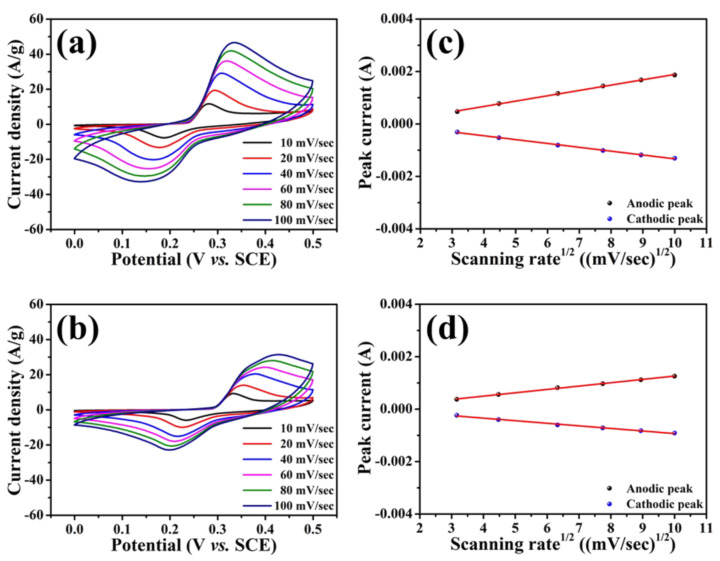
Cyclic voltammograms of and the corresponding anodic/cathodic peak currents as functions of the square root of the scan rates for NiS/A-PVP-NC (**a**,**c**) and NiS/PI-NC (**b**,**d**).

To explore the influence of nitrogen content and structural characteristics on the electrochemical performances of as-synthesized NiS/nitrogen-doped carbon nanocomposites, the cyclic voltammetry was carried out in the potential range between 0 V and 0.5 V (vs. SCE) at a scan rate of 10 mV/sec to 100 mV/sec under an N_2_-saturated 6 M KOH electrolyte. The corresponding cyclic voltammograms (CVs) depicted in Figure 5a,b show that a couple of well-defined redox peaks in each NiS/nitrogen-doped carbon nanocomposite were detected. The resulting curves are distinguishable from the rectangular shape for electric double-layer capacitors, indicating that the energy storage of the NiS electrode is mainly attributed to the reversible Faradic redox reaction of NiS + OH^−^ ↔ NiSOH + e^−^ [56]. Besides, all CV graphs have stable shapes even as the scan rate increased to 100 mV/sec, showing the outstanding reversible reaction and rapid redox processes [57]. Especially highlighted is that current densities outputted from the NiS/A-PVP-NC were superior to those of the NiS/PI-NC among the various scan rates, that is, 29.1 A/g vs. 20.4 A/g at 40 mV/sec (Figure S4a). However, it is apparent that there is a slight shift at oxidation and reduction peaks towards the positive and negative directions of potential when the scan rate rose, indicating that the charge transfer from the electrolyte to electrode materials and vice versa is the rate-determining step at a high scan rate [57]. Considering the potential difference (ΔV) between anodic and cathodic peak positions, the ΔV values of NiS/A-PVP-NC shown in Figure S4b were smaller and even better than those of the NiS/PI-NC, especially at 100 mV/sec, that is, 197 mV vs. 227 mV (vs. SCE). Table S2 also summarizes the detailed values for both samples. The results discussed above can be recognized in the uniform distribution of the NiS onto A-PVP-NC and the better wettability between the NiS/A-PVP-NC and 6 M KOH electrolyte, as proven in Figure 2c and Figure 4.

For further understanding of the storage mechanism, their peak currents as functions of the square root of the scan rates are plotted in Figure 5c,d. Obviously, a shift of the anodic and cathodic peaks to higher (oxidation reaction) and lower (reduction reaction) potentials appeared as the scan rate gradually increased. This refers to the rate-determining step being controlled by the diffusion of electrolyte ions, which implies that both NiS/nitrogen-doped carbon nanocomposites have the capability to store charges as battery-type electrode materials [58]. Encouragingly, the slope of NiS/A-PVP-NC is greater than that of NiS/PI-NC, suggesting rapid diffusion of the electrolyte ions. Moreover, Figure S5 shows the relationship between current density and scan rate based on the following equation: *i* = *kν^b^*, where *i* is the current density (A/g), *v* is the scan rate (V/sec), and *k* and *b* are adjustable parameters. The value of b provides information about the charge storage kinetics. b = 1 represents an ideal capacitive behavior, while b = 0.5 suggests a diffusion controlled process. Such values can be calculated by plotting the logarithm of the absolute current density against the logarithm of the scan rate, that is log *i* = *b* log *v* + *k* [59,60]. The obtained b values of NiS/A-PVP-NC and NiS/PI-NC were calculated to be 0.62 and 0.58 from the cathodic (reduction) peaks, signifying that the dominant process is diffusion controlled. This confirmed again that both electrodes process a battery-like behavior. Once the NiS nanoparticles had contact with the KOH electrolyte, the electroactive species, NiSOH, were produced accordingly. Meanwhile, the amount of such species depends on the contact area of the NiS nanoparticles. Therefore, rapid diffusion of the electrolyte ions could be helpful for a significant increase in capacitance [61].

**Figure 6:**
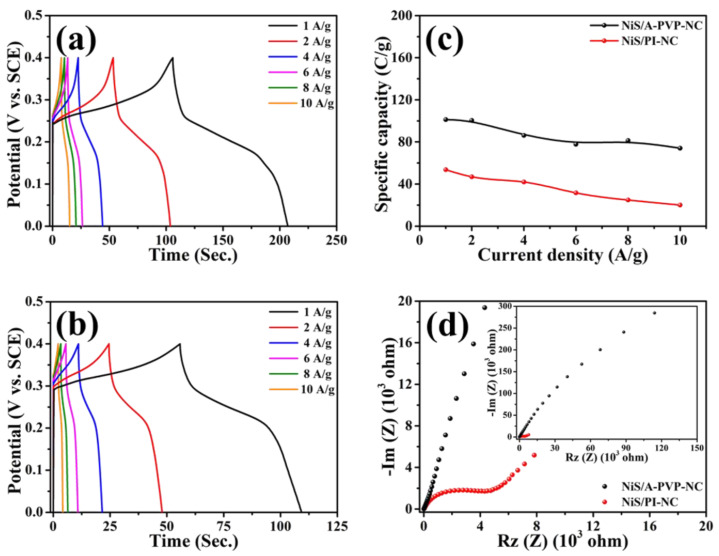
Galvanostatic charge–discharge profiles of NiS/A-PVP-NC (**a**) and NiS/PI-NC (**b**), their specific capacities as functions of the current densities (**c**), and electrochemical impedance spectra (**d**).

Supported by the positive results disclosed in the CVs, the galvanostatic charge–discharge (GCD) tests were conducted in the potential range of 0 V to 0.4 V (vs. SCE) at current densities between 1 A/g to 10 A/g. As illustrated in Figure 6a,b, the charge–discharge plateaus were evidently displayed, and were consistent with the redox potentials obtained in the CV curves. In comparison with the NiS/PI-NC, the specific capacities reflected from the NiS/A-PVP-NC at all current densities were greater. The value recorded at 10 A/g was 3.7 times more than that of NiS/PI-NC, that is, 74 C/g vs. 20 C/g (Figure 6c). This considerable enhancement would not only be supported by a uniform distribution of the NiS onto A-PVP-NC and a larger proportion of the Ni^3+^ observed from the XPS evidence (Figure 3d), but would also be correlated with the better wettability between the NiS/A-PVP-NC and alkaline electrolyte, and with the lower electronic resistance [46] confirmed by EIS spectra (Figure 6d). Furthermore, it is apparent that a semi-circuit at medium frequency was observed from the NiS/PI-NC, which is correlated with the presence of double-layer capacitance due to its low hydrophilic nature [62]. This causes higher charge-transfer resistance as compared with the NiS-A-PVP-NC. In addition, the small diffusion current appeared in the NiS/PI-NC at low frequency as a straight line, which is likely due to the absence of pores and poor wettability between the material surface and the electrolyte, leading to a shorter diffusion pathway [63]. Table 1 compares the capacitive performances of various metal sulfide/carbon nanocomposites. The specific capacities reported in the present study may not be as high as most of those announced in the literature. However, it is worth mentioning that the capacity retention for the NiS/A-PVP-NC was above that of most cases, for example, CoS/g-C_3_N_4_ [45], NiS/N-doped carbon [57], CuS/rGO [64], VS_4_/CNTs/rGO [65], NiS/N-doped graphene [66], Ni_3_S_2_/CNFs [67], and NiS/rGO [68].

## 4. Conclusions

In summary, this study presents a facile and efficient microwave-assisted approach for synthesizing NiS/nitrogen-doped carbon nanocomposites to explore the influence of nitrogen content and structural characteristics of polymer-derived nitrogen-doped carbon materials on the capacitive performance of alkaline supercapacitors. As indicated, the high purity, great crystallinity and hierarchically porous architectures of NiS/A-PVP-NC were successfully acquired by a microwave reactor at 150 °C for 1 h. Nevertheless, the dispersity of NiS nanoparticles varied considerably depending on the different textural properties obtained from the polymer-derived nitrogen-doped carbon materials. Due to the fascinating properties and synergistic features, that is., uniform distribution of the NiS, a higher proportion of Ni^3+^, better wettability, and superior electronic conductivity of NiS/A-PVP-NC, a smaller potential difference and excellent reversibility, rate capability and capacity retention were achieved accordingly. The results shown in this study suggest that the oxygen-containing functional groups and porous architectures within the carbon materials have more dramatic and positive effects on the electrochemical performances of alkaline supercapacitors than does the nitrogen content.

## Figures and Tables

**Table 1:** Comparisons of the capacitive performances on various reported metal sulfide/carbon nanocomposites for supercapacitors.

Electrode Materials	Electrolyte	Specific Capacity ^1^ (C/g)	Capacity Retention ^2^ (%)	References
CoS/g-C_3_N_4_	3 M KOH	301@1 A/g, 207@10 A/g	69	[45]
NiS/porous carbon	2 M KOH	609@1 A/g, 453@10 A/g	74	[47]
NiS/N-doped carbon	6 M KOH	665@1 A/g, 346@10 A/g	52	[57]
CuS/rGO	6 M KOH	235@1 A/g, 122@10 A/g	52	[64]
VS_4_/CNTs/rGO	1 M LiClO_4_/PC	1018@1 A/g, 413@10 A/g	41	[65]
NiS/N-doped graphene	6 M KOH	504@1 A/g, 160@10 A/g	32	[66]
Ni_3_S_2_/CNFs	2 M KOH	97@1 A/g, 60@10 A/g	63	[67]
NiS/rGO	6 M KOH	124@1 A/g, 50@10 A/g	40	[68]
NiCo_2_S_4_/graphene	3 M KOH	297@1 A/g, 279@10 A/g	94	[69]
CoS/graphene	2 M KOH	1354@1 A/g, 1061@10 A/g	78	[70]
NiS/A-PVP-NC	6 M KOH	101@1 A/g, 74@10 A/g	73	This study
NiS/PI-NC	6 M KOH	54@1 A/g, 20@10 A/g	37	This study

^1^ Converting from the F/g by considering the potential window of the galvanostatic charge-discharge test. ^2^ The value obtained by dividing the capacity recorded at 10 A/g to that at 1 A/g.

## Data Availability

No new data were created or analyzed in this study. Data sharing is not applicable to this article.

## Supplementary Materials

The following are available online at https://www.mdpi.com/article/10.3390/nano11071867/s1, Figure S1: Nitrogen adsorption-desorption isotherm of A-PVP-NC collected by an accelerated surface area and porosimetry system at 77 K. Inset shows the pore size distribution curve calculated by BJH model, Figure S2: SEM-DEX mappings of (a) NiS-A-PVP-NC and (b) NiS-PI-NC. Scale bar: 8 um. Figure S3. High-resolution XPS spectra of the NiS/A-PI-NC: (a) C 1s, (b) S 2p, (c) O 1s, (d) Ni 2p and (e) N 1s. Figure S4: (a) Cyclic voltammograms and (b) potential differences between anodic and cathodic peak positions of NiS/A-PVP-NC (•) and NiS/PI-NC (•). Figure S5. Anodic and cathodic current densities (log (*i*)) of (a) NiS/A-PVP-NC and (b) NiS/PI-NC plotted as a function of scan rate (log (*v*)). Table S1: Textural properties of the A-PVP-NC. Table S2: The potential differences (ΔV) between anodic and cathodic peak positions of NiS/A-PVP-NC and NiS/PI-NC.

## References

[1] Truong H.V.A., Dao H.V., Do T.C., Ho C.M., To X.D., Dang T.D., Ahn K.K. (2020). Mapping fuzzy energy management strategy for PEM Fuel Cell–Battery–Supercapacitor hybrid excavator. Energies.

[2] Andrade T.S., Dracopoulos V., Lianos P. (2021). Solar energy conversion and storage using a photocatalytic fuel cell combined with a supercapacitor. Electronics.

[3] Kandambeth S., Kale V.S., Shekhah O., Alshareef H.N., Eddaoudi M. (2021). 2D covalent-organic framework electrodes for supercapacitors and rechargeable metal-ion batteries. Adv. Energy Mater..

[4] Lei W., Liu H., Xiao J., Wang Y., Lin L. (2019). Moss-derived mesoporous carbon as bi-functional electrode materials for lithium–sulfur batteries and supercapacitors. Nanomaterials.

[5] Sambasivam S., Raghavendra K., Yedluri A.K., Arbi H.M., Narayanaswamy V., Gopi C.V., Choi B.-C., Kim H.-J., Alzahmi S., Obaidat I.M. (2021). Facile fabrication of MnCo_2_O_4_/NiO flower-like nanostructure composites with improved energy storage Capacity for High-Performance Supercapacitors. Nanomaterials.

[6] Zhao Y., Shi Z., Li H., Wang C.-A. (2018). Designing pinecone-like and hierarchical manganese cobalt sulfides for advanced supercapacitor electrodes. J. Mater. Chem. A.

[7] Colipai C., Southam G., Oyarzún P., González D., Díaz V., Contreras B., Nancucheo I. (2018). Synthesis of copper sulfide nanoparticles using biogenic H_2_S produced by a low-pH sulfidogenic bioreactor. Minerals.

[8] Ansari S.A., Parveen N., Al-Othoum M.A.S., Ansari M.O. (2021). Effect of Washing on the Electrochemical Performance of a Three-Dimensional Current Collector for Energy Storage Applications. Nanomaterials.

[9] Opra D.P., Gnedenkov S.V., Sinebryukhov S.L., Gerasimenko A.V., Ziatdinov A.M., Sokolov A.A., Podgorbunsky A.B., Ustinov A.Y., Kuryavyi V.G., Mayorov V.Y. (2021). Enhancing Lithium and Sodium Storage Properties of TiO_2_(B) Nanobelts by Doping with Nickel and Zinc. Nanomaterials.

[10] Zhao D., Zhao Q., Wang Z., Feng L., Zhang J., Niu C. (2021). PEDOT-Coated Red Phosphorus Nanosphere Anodes for Pseudocapacitive Potassium-Ion Storage. Nanomaterials.

[11] Zhang L., Hu X., Wang Z., Sun F., Dorrell D.G. (2018). A review of supercapacitor modeling, estimation, and applications: A control/management perspective. Renew. Sustain. Energy Rev..

[12] Muzaffar A., Ahamed M.B., Deshmukh K., Thirumalai J. (2019). A review on recent advances in hybrid supercapacitors: Design, fabrication and applications. Renew. Sustain. Energy Rev..

[13] Rathinamala I., Babu I.M., William J.J., Muralidharan G., Prithivikumaran N. (2021). Extra-Durable Hybrid Supercapacitor Based on Cobalt Sulfide and Carbon (MWCNT) Matrix Electrodes. J. Energy Storage.

[14] Wang T., Liu M., Ma H. (2017). Facile synthesis of flower-like copper-cobalt sulfide as binder-free faradaic electrodes for supercapacitors with improved electrochemical properties. Nanomaterials.

[15] Liu Q., Hong X., You X., Zhang X., Zhao X., Chen X., Ye M., Liu X. (2020). Designing heterostructured metal sulfide core-shell nanoneedle films as battery-type electrodes for hybrid supercapacitors. Energy Storage Mater..

[16] Li H., Li Z., Wu Z., Sun M., Han S., Cai C., Shen W., Fu Y. (2019). Nanocomposites of cobalt sulfide embedded carbon nanotubes with enhanced supercapacitor performance. J. Electrochem. Soc..

[17] Wang X., Tian L., Long X., Yang M., Song X., Xie W., Liu D., Fu Y., Li J., Li Y. (2021). Cracked bark-inspired ternary metallic sulfide (NiCoMnS_4_) nanostructure on carbon cloth for high-performance aqueous asymmetric supercapacitors. Sci. China Mater..

[18] Chang R.-J., Sheng Y., Chen T., Mkhize N., Lu Y., Bhaskaran H., Warner J.H. (2019). Morphology Control of Two-Dimensional Tin Disulfide on Transition Metal Dichalcogenides Using Chemical Vapor Deposition for Nanoelectronic Applications. ACS Appl. Nano Mater..

[19] Sridhar V., Park H. (2018). Carbon nanofiber linked FeS_2_ mesoporous nano-alloys as high capacity anodes for lithium-ion batteries and supercapacitors. J. Alloys Compd..

[20] Attia S.Y., Mohamed S.G., Barakat Y.F., Hassan H.H., Zoubi W.A. (2021). Supercapacitor electrode materials: Addressing challenges in mechanism and charge storage. Rev. Inorg. Chem..

[21] Hillier N., Yong S., Beeby S. (2020). The good, the bad and the porous: A review of carbonaceous materials for flexible supercapacitor applications. Energy Rep..

[22] Li Z., Peng H., Liu R., Mo Y., Cao B., Lai W., Li X., Pan L., Chen Y. (2020). Quantitative assessment of basal-, edge- and defect-surfaces of carbonaceous materials and their influence on electric double-layer capacitance. J. Power Sources.

[23] Han X., Jiang H., Zhou Y., Hong W., Zhou Y., Gao P., Ding R., Liu E. (2018). A high performance nitrogen-doped porous activated carbon for supercapacitor derived from pueraria. J. Alloys Compd..

[24] Mostazo-López M.J., Ruiz-Rosas R., Castro-Muñiz A., Nishihara H., Kyotani T., Morallón E., Cazorla-Amorós D. (2018). Ultraporous nitrogen-doped zeolite-templated carbon for high power density aqueous-based supercapacitors. Carbon.

[25] Zuliani J.E., Tong S., Jia C.Q., Kirk D.W. (2018). Contribution of surface oxygen groups to the measured capacitance of porous carbon supercapacitors. J. Power Sources.

[26] Sarkar A., Chakraborty A.K., Bera S., Krishnamurthy S. (2018). Novel hydrothermal synthesis of CoS_2_/MWCNT nanohybrid electrode for supercapacitor: A systematic investigation on the influence of MWCNT. J. Phys. Chem. C.

[27] Yang S., Huang P., Duan M., Li Y., Gao G. (2019). Controllable Synthesis of Iron Sulfide/CNT Nanocomposites in Solvothermal System. Cryst. Res. Technol..

[28] Choi S.H., Kang Y.C. (2015). Aerosol-assisted rapid synthesis of SnS-C composite microspheres as anode material for Na-ion batteries. Nano Res..

[29] Zhou S., Huang Y., Xu L., Zheng W. (2018). Microwave-assisted synthesis of graphene-NiS/Ni_3_S_2_ composites for enhanced microwave absorption behaviors through a sulfuration method. Ceram. Int..

[30] Bahmani M., Imani M., Tadjarodi A. (2021). Facile Microwave-Assisted Preparation of Hetero-Structured CuCo_2_S_4_/CuCo_2_O_4_ Nanoparticles Using Organic Agent of Thiourea. Chem. Proc..

[31] He M., Zhou Y., Huang T., Nie S., Wang Y., Xu Z., Huo Y., Xu R., Chen X., Peng H. (2020). Flower-like CoS hierarchitectures@polyaniline organic-inorganic heterostructured composites: Preparation and enhanced microwave absorption performance. Compos. Sci. Technol..

[32] Scibioh M.A., Viswanathan B., Scibioh M.A., Viswanathan B. (2020). Chapter 3—Electrode materials for supercapacitors. Materials for Supercapacitor Applications.

[33] Algadri N.A., Ibrahim K., Hassan Z., Bououdina M. (2017). Cost-effective single-step carbon nanotube synthesis using microwave oven. Mater. Res. Express.

[34] Sevilla M., Díez N., Fuertes A.B. (2021). More Sustainable Chemical Activation Strategies for the Production of Porous Carbons. ChemSusChem.

[35] Lee H.-M., Kim K.-W., Park Y.-K., An K.-H., Park S.-J., Kim B.-J. (2019). Activated Carbons from Thermoplastic Precursors and Their Energy Storage Applications. Nanomaterials.

[36] Vijayakumar S., Nagamuthu S., Ryu K.-S. (2017). CuCo_2_O_4_ flowers/Ni-foam architecture as a battery type positive electrode for high performance hybrid supercapacitor applications. Electrochim. Acta.

[37] Subramanyam P., Ghosal P., Deepa M., Subrahmanyam C. (2018). Cuprous Sulfide@Carbon nanostructures based counter electrodes with cadmium sulfide/titania photoanode for liquid junction solar cells. Electrochim. Acta.

[38] Vatankhah A.R., Hosseini M.A., Malekie S. (2019). The characterization of gamma-irradiated carbon-nanostructured materials carried out using a multi-analytical approach including Raman spectroscopy. Appl. Surf. Sci..

[39] Hung T.-F., Cheng W.-J., Chang W.-S., Yang C.-C., Shen C.-C., Kuo Y.-L. (2016). Ascorbic Acid-Assisted Synthesis of Mesoporous Sodium Vanadium Phosphate Nanoparticles with Highly sp^2^-Coordinated Carbon Coatings as Efficient Cathode Materials for Rechargeable Sodium-Ion Batteries. Chem. Eur. J..

[40] Zolkin A., Semerikova A., Chepkasov S., Khomyakov M. (2017). Characteristics of the Raman spectra of diamond-like carbon films. Influence of methods of synthesis. Mater. Today Proc..

[41] Xiong B., Li J., He C., Tang X., Lv Z., Li X., Yan X. (2020). Effect of pore morphology and surface roughness on wettability of porous titania films. Mater. Res. Express.

[42] Kim Y.K., Park H.B., Lee Y.M. (2005). Gas separation properties of carbon molecular sieve membranes derived from polyimide/polyvinylpyrrolidone blends: Effect of the molecular weight of polyvinylpyrrolidone. J. Membr. Sci..

[43] Abdelaal M.M., Mohamed S., Barakat Y.F., Derbala H.A.Y., Hassan H.H., Al Zoubi W. (2018). N-Aminophthalimide as a Synthon for Heterocyclic Schiff bases: Efficient Utilization as Corrosion Inhibitors of Mild Steel in 0.5 mol·L^−1^ H_2_SO_4_ Solution. Egypt. J. Chem..

[44] Geiszler V.C., Koros W.J. (1996). Effects of Polyimide Pyrolysis Conditions on Carbon Molecular Sieve Membrane Properties. Ind. Eng. Chem. Res..

[45] Jiang D., Xu Q., Meng S., Xia C., Chen M. (2017). Construction of cobalt sulfide/graphitic carbon nitride hybrid nanosheet composites for high performance supercapacitor electrodes. J. Alloys Compd..

[46] Hung T.-F., Yin Z.-W., Betzler S.B., Zheng W., Yang J., Zheng H. (2019). Nickel sulfide nanostructures prepared by laser irradiation for efficient electrocatalytic hydrogen evolution reaction and supercapacitors. Chem. Eng. J..

[47] Wu J., Wei F., Sui Y., Qi J., Zhang X. (2020). Interconnected NiS-nanosheets@porous carbon derived from Zeolitic-imidazolate frameworks (ZIFs) as electrode materials for high-performance hybrid supercapacitors. Int. J. Hydrogen Energy.

[48] Wagner C.D., Naumkin A.V., Kraut-Vass A., Allison J.W., Powell C.J., Rumble J.R. NIST X-ray Photoelectron Spectroscopy Database. https://srdata.nist.gov/xps/selEnergyType.aspx.

[49] Zhang L., Tu L.-Y., Liang Y., Chen Q., Li Z.-S., Li C.-H., Wang Z.-H., Li W. (2018). Coconut-based activated carbon fibers for efficient adsorption of various organic dyes. RSC Adv..

[50] Zhao J., Guan B., Hu B., Xu Z., Wang D., Zhang H. (2017). Vulcanizing time controlled synthesis of NiS microflowers and its application in asymmetric supercapacitors. Electrochim. Acta.

[51] Lazar P., Mach R., Otyepka M. (2019). Spectroscopic fingerprints of graphitic, pyrrolic, pyridinic, and chemisorbed nitrogen in N-doped graphene. J. Phys. Chem. C.

[52] Hung T.-F., Hsieh T.-H., Tseng F.-S., Wang L.-Y., Yang C.-C., Yang C.-C. (2021). High-Mass Loading Hierarchically Porous Activated Carbon Electrode for Pouch-Type Supercapacitors with Propylene Carbonate-Based Electrolyte. Nanomaterials.

[53] Le T., Yang Y., Huang Z., Kang F. (2015). Preparation of microporous carbon nanofibers from polyimide by using polyvinyl pyrrolidone as template and their capacitive performance. J. Power Sources.

[54] Zhao Y., Wang H., Liu J., Liu J., Li G., Peng H., Chen K., Zhang Z. (2020). Nitrogen-and Oxygen-Containing Three-Dimensional Hierarchical Porous Graphitic Carbon for Advanced Supercapacitor. Nanomaterials.

[55] Yan G., Bai L., Feng J., Zhang Z. (2020). A Comparative Study on the Wettability of Two Coal Samples during Deep Burial Metamorphism. J. Chem..

[56] Yedluri A.K., Kulurumotlakatla D.K., Sangaraju S., Ihab M.O., Kim H.-J. (2020). Facile synthesis of novel and highly efficient CoNi_2_S_4_-Ni(OH)_2_ nanosheet arrays as pseudocapacitive-type electrode material for high-performance electrochemical supercapacitors. J. Energy Storage.

[57] Zhu H., Sun X., Yang H., Ta S., Wang L., Zhu H., Zhang Q. (2021). Polydopamine-derived nitrogen-doped carbon-coated NiS nanoparticles as a battery-type electrode for high-performance supercapacitors. Ceram. Int..

[58] Wu Z., Huang H., Xiong W., Yang S., Huang H., Zou Y., Zhou W., Cheng Z., Wang J., Luo G. (2021). One-Pot Synthesis of Glucose-Derived Carbon Coated Ni_3_S_2_ Nanowires as a Battery-Type Electrode for High Performance Supercapacitors. Nanomaterials.

[59] Yao B., Peng H., Zhang H., Kang J., Zhu C., Delgado G., Byrne D., Faulkner S., Freyman M., Lu X. (2021). Printing porous carbon aerogels for low temperature supercapacitors. Nano Lett..

[60] Yao B., Chandrasekaran S., Zhang H., Ma A., Kang J., Zhang L., Lu X., Qian F., Zhu C., Duoss E.B. (2020). 3D-Printed Structure Boosts the Kinetics and Intrinsic Capacitance of Pseudocapacitive Graphene Aerogels. Adv. Mater..

[61] Theerthagiri J., Thiagarajan K., Senthilkumar B., Khan Z., Senthil R.A., Arunachalam P., Madhavan J., Ashokkumar M. (2017). Synthesis of hierarchical cobalt phosphate nanoflakes and their enhanced electrochemical performances for supercapacitor applications. ChemistrySelect.

[62] He Y., Zhang Y., Li X., Lv Z., Wang X., Liu Z., Huang X. (2018). Capacitive mechanism of oxygen functional groups on carbon surface in supercapacitors. Electrochim. Acta.

[63] An G.-H. (2020). Ultrafast long-life zinc-ion hybrid supercapacitors constructed from mesoporous structured activated carbon. Appl. Surf. Sci..

[64] El-Hout S.I., Mohamed S.G., Gaber A., Attia S.Y., Shawky A., El-Sheikh S.M. (2021). High electrochemical performance of rGO anchored CuS nanospheres for supercapacitor applications. J. Energy Storage.

[65] Wang X., Zhang Y., Zheng J., Jiang H., Dong X., Liu X., Meng C. (2020). Fabrication of vanadium sulfide (VS_4_) wrapped with carbonaceous materials as an enhanced electrode for symmetric supercapacitors. J. Colloid Interface Sci..

[66] Reddy B.J., Vickraman P., Justin A.S. (2019). Electrochemical performance of nitrogen-doped graphene anchored nickel sulfide nanoflakes for supercapacitors. Appl. Surf. Sci..

[67] Yu W., Lin W., Shao X., Hu Z., Li R., Yuan D. (2014). High performance supercapacitor based on Ni_3_S_2_/carbon nanofibers and carbon nanofibers electrodes derived from bacterial cellulose. J. Power Sources.

[68] Marand N.A., Masoudpanah S., Alamolhoda S., Bafghi M.S. (2021). Solution combustion synthesis of nickel sulfide/reduced graphene oxide composite powders as electrode materials for high-performance supercapacitors. J. Energy Storage.

[69] Zhao F., Xie D., Huang W., Song X., Sial M.A.Z.G., Wu H., Deng F., Zhang Q., Zou J., Zeng X. (2020). Defect-rich honeycomb-like nickel cobalt sulfides on graphene through rapid microwave-induced synthesis for ultrahigh rate supercapacitors. J. Colloid Interface Sci..

[70] Shi J., Li X., He G., Zhang L., Li M. (2015). Electrodeposition of high-capacitance 3D CoS/graphene nanosheets on nickel foam for high-performance aqueous asymmetric supercapacitors. J. Mater. Chem. A.

