# Supplementary material for: A Comparative Study of the Influence of Nitrogen Content and Structural Characteristics of NiS/Nitrogen-Doped Carbon Nanocomposites on Capacitive Performances in Alkaline Medium
Source: Nanomaterials (Basel). 2021 Jul 20;11(7):1867. doi: 10.3390/nano11071867 (PMC8308313; doi:10.3390/nano11071867)
Supplement: Supplementary file 1 [file nanomaterials-11-01867-s001.zip › nanomaterials-1297559-supplementary.pdf]

nanomaterials

Supplementary Materials

A Comparative Study of the Influence of Nitrogen Content and Structural Characteristics of NiS/Nitrogen-Doped Carbon Nanocomposites on Capacitive Performances in Alkaline Medium

Mohamed M. Abdelaal [1,2,†], Tzu-Cheng Hung [1,†], Saad Gomaa Mohamed [2], Chun-Chen Yang [1,3,4], Huei-Ping Huang [1] and Tai-Feng Hung [1,*]

1 Battery Research Center of Green Energy, Ming Chi University of Technology, 84 Gungjuan Rd., Taishan District, New Taipei City 24301, Taiwan; mohamedbec@yahoo.com (M.M.A.); u07137023@mail2.mcut.edu.tw (T.-C.H.); ccyang@mail.mcut.edu.tw (C.-C.Y.); cindy118036@mail.mcut.edu.tw (H.-P.H.)
2 Tabbin Institute for Metallurgical Studies (TIMS), Tabbin, Helwan 109, Cairo 11421, Egypt; sgmmohamed@gmail.com
3 Department of Chemical Engineering, Ming Chi University of Technology, 84 Gungjuan Rd., Taishan District, New Taipei City 24301, Taiwan
4 Department of Chemical and Materials Engineering, Chang Gung University, 259 Wenhua 1st Rd., Guishan District, Taoyuan 33302, Taiwan
* Correspondence: taifeng@mail.mcut.edu.tw; Tel.: +886-2-2908-9899 (etx. 4957)
† These authors contributed to this work equally.

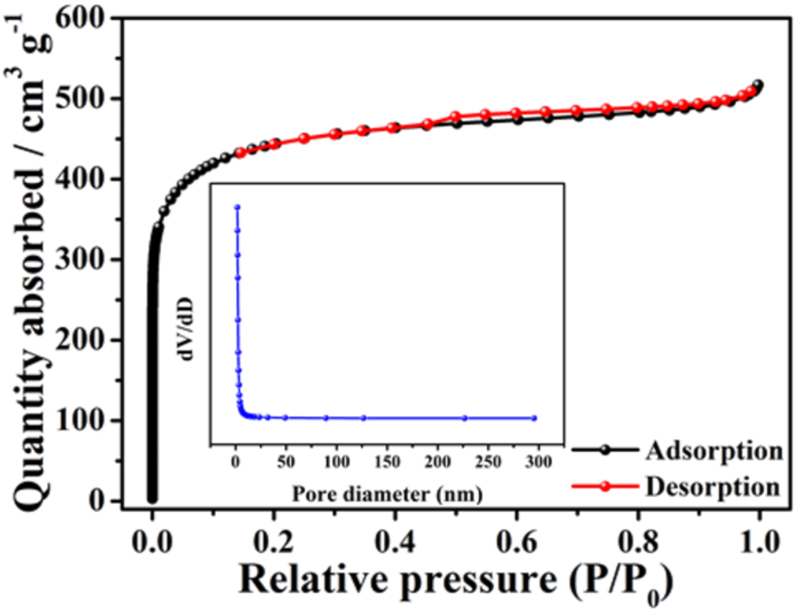

Figure S1. Nitrogen adsorption-desorption isotherm of A-PVP-NC collected by an accelerated surface area and porosimetry system at 77 K. Inset shows the pore size distribution curve calculated by BJH model.

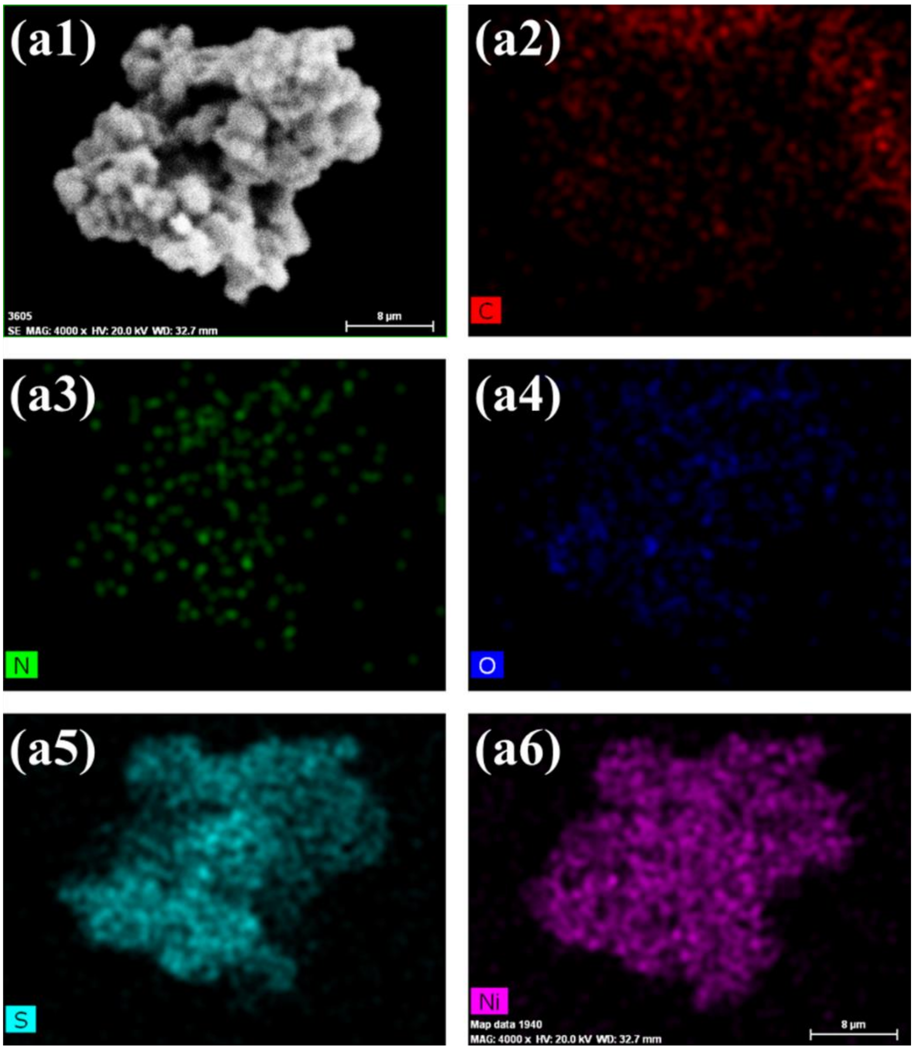
(a1)
3605
SE MAG: 4000 x HV: 20.0 kV WD: 32.7 mm
8 µm
(a2)
C
(a3)
N
(a4)
O
(a5)
S
(a6)
Ni
Map data 1940
MAG: 4000 x HV: 20.0 kV WD: 32.7 mm
8 µm

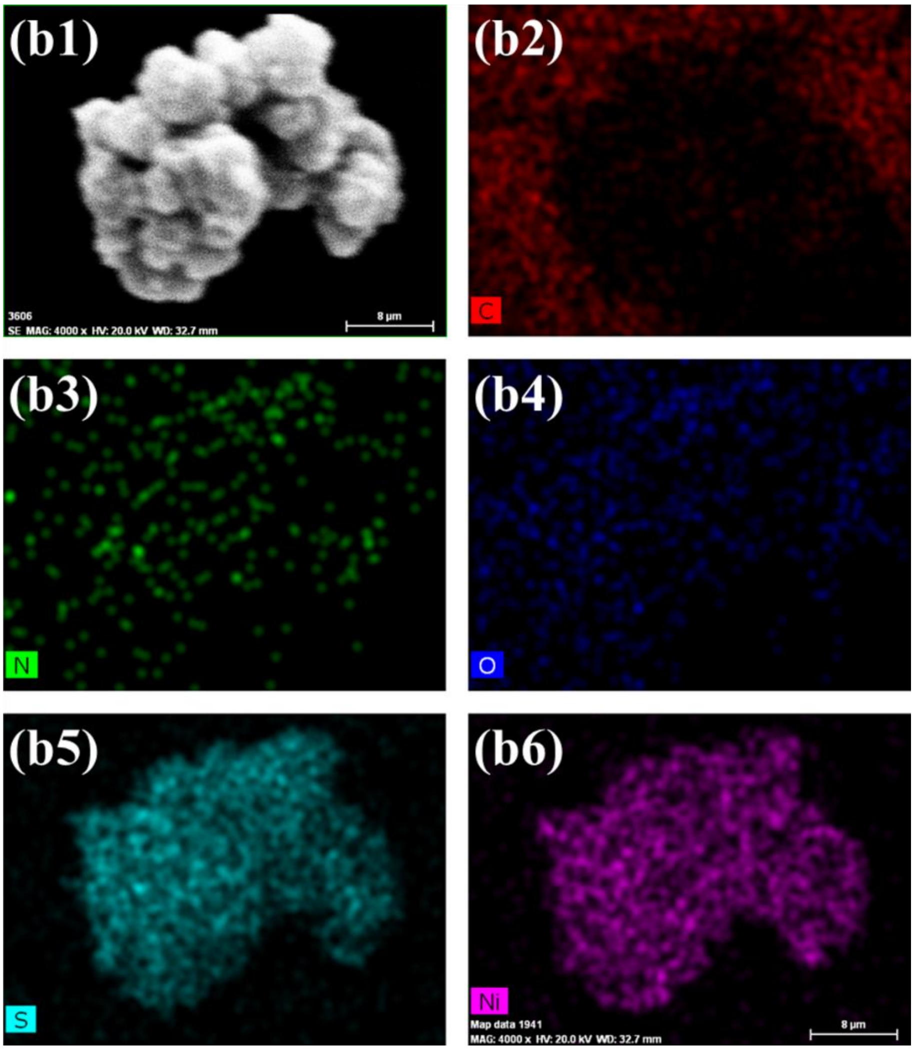

Figure S2. SEM-DEX mappings of (**a**) NiS-A-PVP-NC: (a1) SEM micrograph, (a2) carbon mapping, (a3) nitrogen mapping, (a4) oxygen mapping, (a5) sulfur mapping, (a6) nickel mapping and (**b**) NiS-PI-NC: (b1) SEM micrograph, (b2) carbon mapping, (b3) nitrogen mapping, (b4) oxygen mapping, (b5) sulfur mapping, (b6) nickel mapping. Scale bar: 8 um.

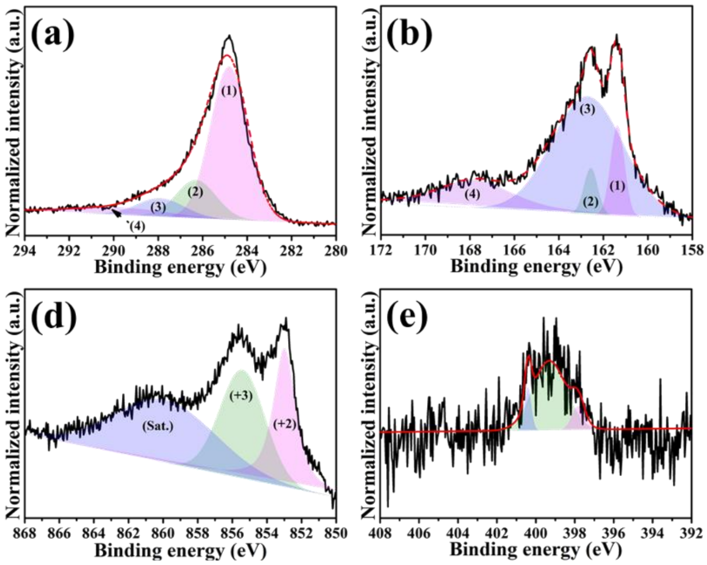

Figure S3. High-resolution XPS spectra of the NiS/A-PI-NC: (**a**) C 1s ((1) for C=C bond, (2) for C−O bond, (3) for C=O bond, and (4) for O=C−O bond), (**b**) S 2p ((1)-(3) assigned to S^{2-} within the NiS, and (4) from sulfate ions), (**c**) O 1s ((1) and (3) are associated with the $Ni^{3+}OOH$ and $Ni^{2+}SO_4$, (2) for O=C−O bond, and (4) for C=O bond), (**d**) Ni 2p and (**e**) N 1s.

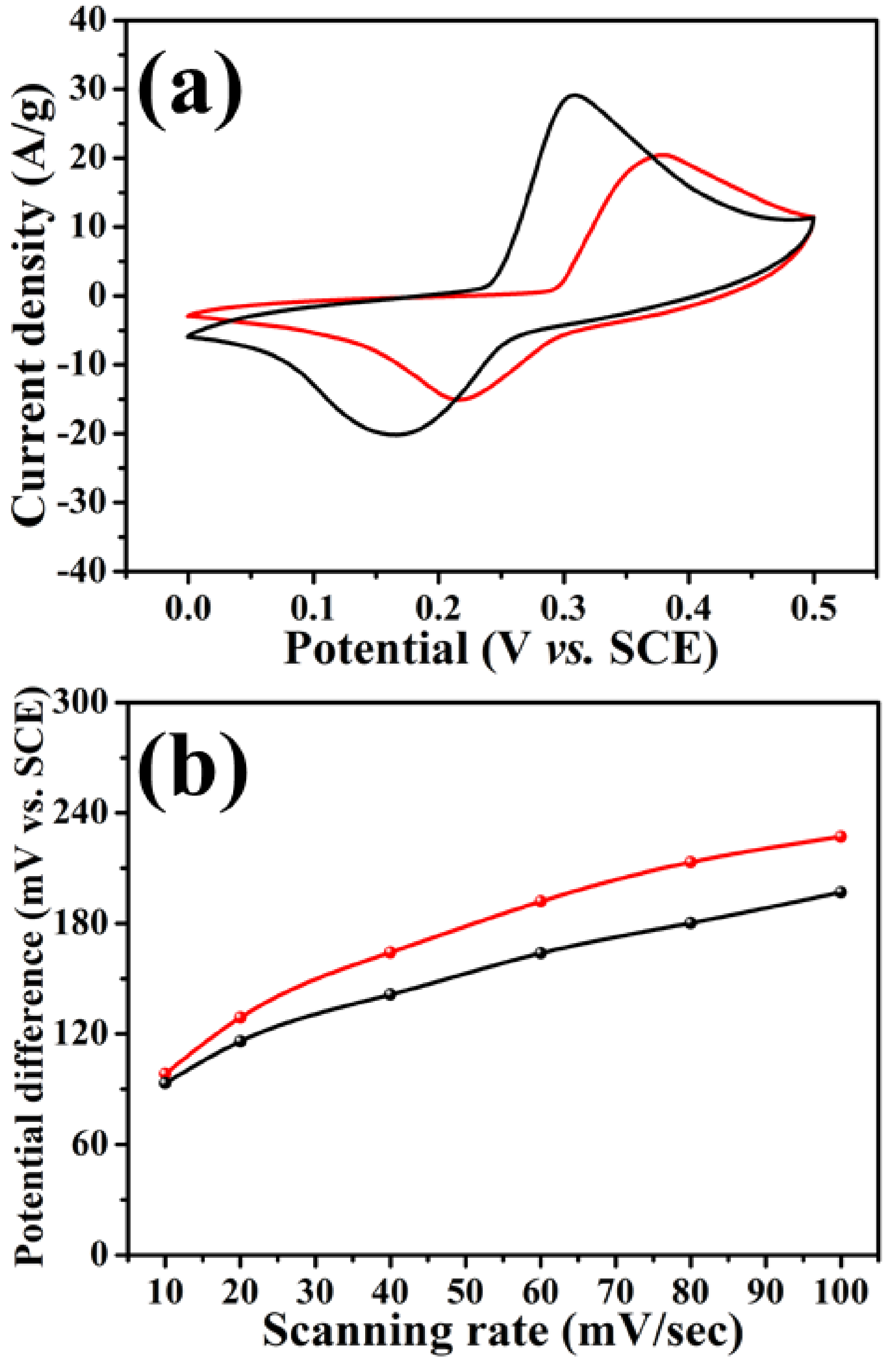

Figure S4. (**a**) Cyclic voltammograms of NiS/A-PVP-NC (-) and NiS/PI-NC (-), and (**b**) potential differences between anodic and cathodic peak positions of NiS/A-PVP-NC (•) and NiS/PI-NC (•).

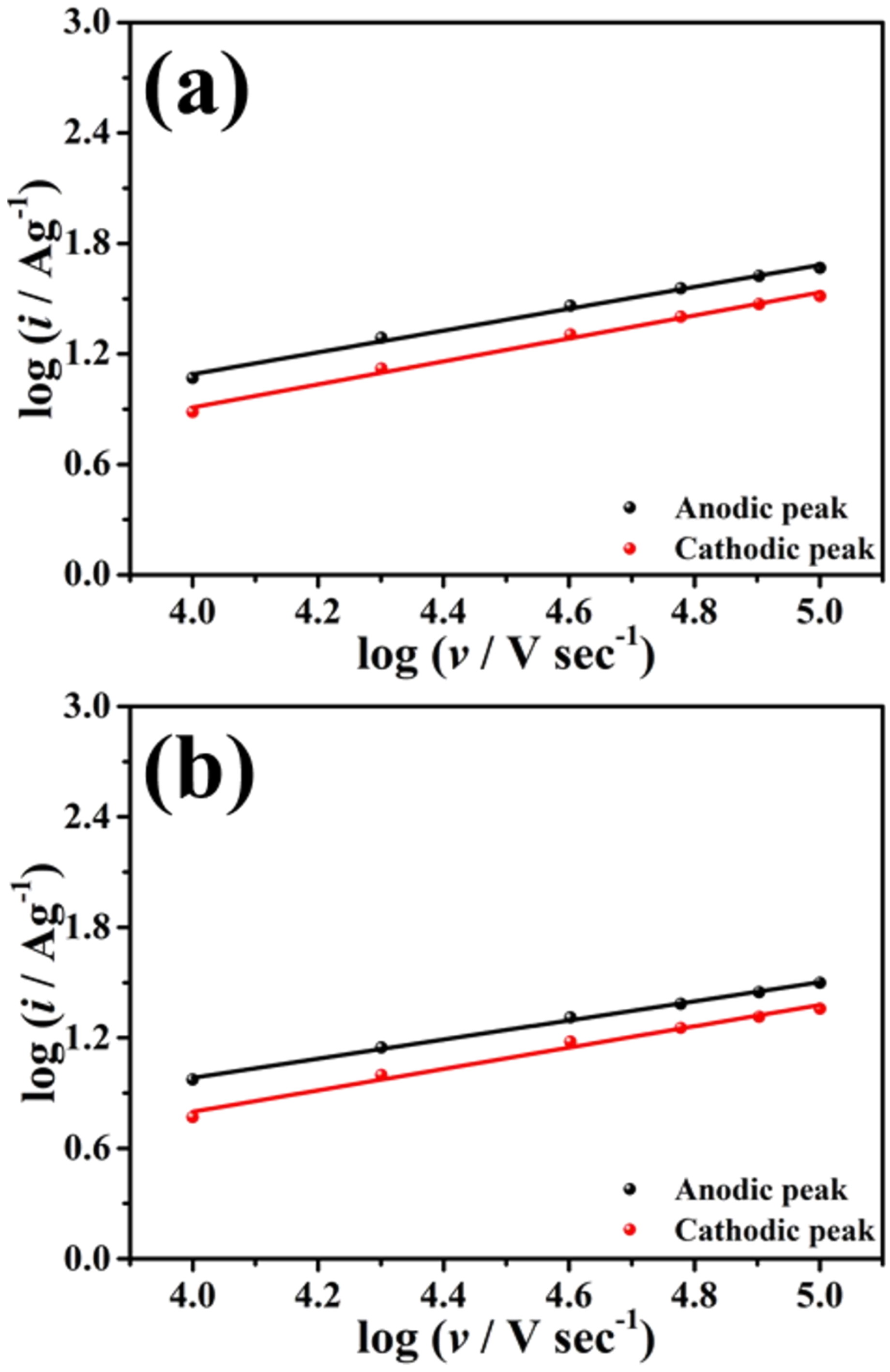

Figure S5. Anodic and cathodic current densities (log (*i*)) of (**a**) NiS/A-PVP-NC and (**b**) NiS/PI-NC plotted as a function of scan rate (log (*v*)).

Table S1. Textural properties of the A-PVP-NC.

Sample	SSA[1] (m^2/g)	V_t[2] (cm^3/g)	V_{ultra}[3] (cm^3/g)	V_{micro}[4] (cm^3/g)	Vmeso[5] (cm^3/g)
A-PVP-NC	1628	0.79	0.25	0.62	0.17

[1]SSA: Specific surface area calculated by the Brunauer-Emmett-Teller method.

[2]V_t: total pore (single-point) volume obtained from the amount of adsorbed nitrogen at $P/P_0 = 0.995$.

[3]V_{ultra}: volume of ultramicropores (pores < 0.7 nm).

[4]V_{micro}: volume of micropores (pores < 2.0 nm).

[5]V_{meso}: volume of mesopores (difference between V_t and V_{micro}).

Table S2. The potential differences (ΔV) between anodic and cathodic peak positions of NiS/A-PVP-NC and NiS/PI-NC.

Scan rate (mV/sec)	ΔV for NiS/A-PVP-NC (mV *vs.* SCE)	ΔV for NiS/PI-NC (mV *vs.* SCE)
10	93.2	98.3
20	116.0	129.0
40	141.5	164.3
60	163.9	192.0
80	180.3	213.4
100	197.1	227.1

© 2021 by the authors. Licensee MDPI, Basel, Switzerland. This article is an open access article distributed under the terms and conditions of the Creative Commons Attribution (CC BY) license (http://creativecommons.org/licenses/by/4.0/).
